# Distinct roles of STAT3 and STAT5 in CAR T cell therapy

**DOI:** 10.1016/j.omton.2026.201224

**Published:** 2026-05-13

**Authors:** Yuki Kagoya

**Affiliations:** 1 Division of Tumor Immunology, Institute for Advanced Medical Research, Keio University School of Medicine, Tokyo, Japan

## Main text

Cytokine signaling is a critical component of adoptive immunotherapy, including CAR T cell therapy. Because large numbers of *ex vivo*-expanded T cells are infused into patients, endogenous cytokine levels are often inadequate to support their optimal survival and function. The most extensively studied cytokines signal through the common γ-chain receptor, which primarily triggers downstream signaling through STAT3 and STAT5.^1^ However, because each cytokine activates multiple STAT proteins simultaneously, it is difficult to dissect the individual roles of STAT proteins in cytokine engineering approaches.

In our recent study, “constitutive STAT3 signaling, in comparison to STAT5, enhances CAR T cell efficacy and lowers systemic toxicity” published in *Cancer Immunology Research*, we separately analyzed the roles of STAT3 and STAT5 signaling in CAR T cell function using constitutively active forms of STAT3 and STAT5 (caSTAT3 and caSTAT5).^2^ Unexpectedly, STAT3 activation induced much broader transcriptional changes than STAT5 activation. The caSTAT3-expressing CAR T cells upregulated genes associated with both memory and effector T cell states. Although we did not perform single-cell RNA sequencing, flow cytometric analysis suggested that these changes did not merely reflect distinct memory-like and effector-like subpopulations. Instead, caSTAT3-expressing CAR T cells appeared to acquire a hybrid state, simultaneously expressing memory- and effector-associated features. These cells showed increased granzyme B production and an IFNγ-dominant monofunctional profile, with reduced IL-2 and TNF-α characteristic of terminal effector differentiation, while retaining higher expression of early memory markers such as CCR7 and CD62L.

Despite these phenotypic changes, constitutive STAT3 activation markedly impaired CAR T cell survival *in vitro*. The caSTAT3-expressing CAR T cells rapidly lost proliferative capacity and underwent apoptosis within a week after transduction. In contrast, despite only modest changes in gene expression, caSTAT5-expressing CAR T cells exhibited prolonged survival and robust proliferative capacity in both the presence and absence of exogenous cytokine supplementation.

These findings were further supported by CRISPR/Cas9-mediated knockout studies. STAT3-ablated CAR T cells exhibited reduced memory phenotypes and granzyme B production. In contrast, STAT3 ablation enhanced cytokine production, particularly IL-2 and TNF-α, consistent with the diminished polyfunctionality observed in caSTAT3-expressing cells. Thus, STAT3 appeared to promote a terminal effector-like functional profile characterized by enhanced cytotoxicity but reduced polyfunctional cytokine production, while simultaneously maintaining memory-associated features.

Whereas STAT5 ablation, as expected, markedly impaired T cell proliferative capacity, STAT3 ablation did not alter antigen-dependent proliferation. These findings suggest that proliferation, cytokine secretion, and cytolytic granule production are governed by distinct transcriptional programs rather than coordinately regulated as a single effector module.

However, the deleterious effect of caSTAT3 on CAR T cell survival was markedly attenuated *in vivo*. caSTAT3-expressing CAR T cells persisted *in vivo* at levels comparable to control CAR T cells while maintaining the enhanced cytotoxic phenotype observed *in vitro*, ultimately resulting in significantly improved tumor control. We confirmed the superior antitumor efficacy of caSTAT3 CAR T cells in both hematologic and solid tumor models.

In the solid tumor model, caSTAT3 CAR T cells progressively accumulated at the tumor site. RNA sequencing (RNA-seq) analysis revealed that these tumor-infiltrating CAR T cells no longer retained the typical memory-associated transcriptional program; instead, they exhibited a pronounced effector profile characterized by the upregulation of genes such as *GZMB*, *PRF1*, and *BATF*.

Another notable feature of caSTAT3 CAR T cells was the alteration of exhaustion-associated programs. The caSTAT3 CAR T cells exhibited reduced expression of exhaustion-associated transcription factors, including TOX and NR4A1-3, together with several inhibitory receptors such as TIGIT and BTLA.

Interestingly, caSTAT3 expression in CAR T cells induced upregulation of TOX2 while downregulating TOX. Although TOX and TOX2 are often discussed together as exhaustion-associated transcription factors, recent studies suggest that TOX2 preferentially supports memory-like and persistent CAR T cell states, whereas TOX is more closely associated with terminal dysfunction and cytokine loss.^3^ Thus, the reciprocal regulation of TOX2 and TOX may underlie the unique phenotype of caSTAT3 CAR T cells, which combined memory-associated features and *in vivo* persistence with terminal effector-like cytotoxic function.

One possible explanation for the discrepant effects of caSTAT3 on CAR T cell survival *in vitro* and *in vivo* is the difference in its expression level. The caSTAT3 was expressed at substantially lower levels *in vivo* than *in vitro*. Consistent with this, partial deletion of the promoter reduced caSTAT3 expression and restored CAR T cell survival *in vitro*, while preserving its memory- and effector-like properties. These findings indicate that the beneficial effects of caSTAT3 depend on achieving an optimal range of expression.

The effects of STAT3 on T cell effector function appear to be bidirectional. Although STAT3 plays an essential role in memory T cell formation,^4^ it has also been widely discussed as an immunosuppressive signal in the context of cancer immunotherapy.^5^ Indeed, STAT3 inhibition has been explored as a strategy to enhance antitumor immunity. This concept is partly explained by the anti-apoptotic effects of STAT3 in tumor cells. In addition, STAT3 activation in myeloid cells promotes the acquisition of immunosuppressive phenotypes. Owing to these multifaceted roles, the direct impact of STAT3 on the antitumor activity of cytotoxic T cells has remained incompletely understood. Our study clearly elucidates the unique effects of STAT3 signaling on effector and memory functions when activated intrinsically in T cells.

Although STAT5 activation extended survival of CAR T cells without significantly affecting cytotoxic function itself, some mice treated with caSTAT5-CAR T cells developed progressive weight loss and subsequently died from non-relapse causes. Autopsy analysis exhibited infiltration of caSTAT5 CAR T cells in multiple organs, suggesting that antigen-independent activation of STAT5 CAR T cells. This effect seems to be elicited by xenogeneic reaction because T cell expansion was markedly reduced by genetic ablation of *TRAC*. However, these data raise an important caution regarding the use of constitutive STAT5 activation in CAR T cells. In solid tumors, truly tumor-specific antigens are rare, and most tumor-associated antigens are expressed to some extent in normal tissues. Thus, the enhanced persistence and tissue infiltration induced by caSTAT5 may increase the risk of on-target, off-tumor toxicity. An open question is how STAT5 activation enhances T cell-mediated immunopathology, particularly because, as observed in our *in vitro* experiments, we could not clearly identify the gene expression changes induced by STAT5 activation.

Finally, we addressed what happens to T cells when STAT3 and STAT5 were simultaneously upregulated.

We found that engineering of both caSTAT3 and caSTAT5 provided CAR T cells with extraordinary long-lived potential. They continued to proliferate, although not infinite, even in the absence of antigen restimulation or exogenous cytokine support. An intriguing finding from the RNA-seq analysis was that the gene expression profile of caSTAT3+caSTAT5 CAR T cells could not be explained by the additive effects of caSTAT3 and caSTAT5 alone. Most cytokines simultaneously activate multiple STAT proteins to different extents, and the resulting combinatorial signaling may underlie their non-redundant functions.

Our study highlights the importance of elucidating the functional roles of individual STAT proteins (Table 1). Recent studies have demonstrated beneficial effects of previously underappreciated cytokines, including IL-4, IL-23, and IL-27, in adoptive cell therapy.^6^ Because these cytokines activate additional STAT proteins, such as STAT1, STAT4, and STAT6, future studies should extend this framework to other STAT family members.

**Table 1:** Roles of STAT3 and STAT5 in CAR T cell function

	STAT3	STAT5
Memory phenotype	↑	↔
Effector function	↑	↔
Antigen-independent survival	↔ ↓ with hyperactivation	↑ (potential toxicity concern)
Major cytokines	IL-2, IL-7, IL-9, IL-15	IL-21, IL-10, IL-23

From a translational perspective, vector design will be critical, as the effects of STAT modulation depend strongly on expression level. Another important consideration is the precise evaluation of the risk of malignant transformation associated with constitutive activation of the JAK-STAT pathway. Although our study confirmed that activation of STAT3 and/or STAT5 alone was insufficient to transform peripheral blood T cells, constitutive JAK-STAT signaling may still cooperate with additional oncogenic alterations, such as TP53 or TET2 loss.^7^

## Declaration of interests

Y.K. received research funding from Takara Bio, Inc. and AGC, Inc. outside the submitted work.

## Declaration of generative AI and AI-assisted technologies in the writing process

During the preparation of this work, the author used ChatGPT for English language editing. After using this tool, the author reviewed and edited the content as needed and takes full responsibility for the content of the published article.
